# Effect of oral glycine on the clinical, spirometric and inflammatory status in subjects with cystic fibrosis: a pilot randomized trial

**DOI:** 10.1186/s12890-017-0528-x

**Published:** 2017-12-15

**Authors:** Mario H. Vargas, Rosangela Del-Razo-Rodríguez, Amando López-García, José Luis Lezana-Fernández, Jaime Chávez, María E. Y. Furuya, Juan Carlos Marín-Santana

**Affiliations:** 1 Departamento de Investigación en Hiperreactividad Bronquial, Instituto Nacional de Enfermedades Respiratorias Ismael Cosío Villegas, Tlalpan 4502, CP 14080 Mexico City, Mexico; 2 Unidad de Investigación Médica en Enfermedades Respiratorias, Hospital de Pediatría, Centro Médico Nacional Siglo XXI, Instituto Mexicano del Seguro Social, Mexico City, Mexico; 3 Servicio Clínico de Neumopediatría, Instituto Nacional de Enfermedades Respiratorias Ismael Cosío Villegas, Mexico City, Mexico; 4 Departamento de Neumología, Hospital de Pediatría, Centro Médico Nacional Siglo XXI, Instituto Mexicano del Seguro Social, Mexico City, Mexico; 5 Laboratorio de Fisiología Pulmonar y Clínica de Fibrosis Quística, Hospital Infantil de México, Mexico City, Mexico; 6 Asociación Mexicana de Fibrosis Quística AC, Mexico City, Mexico; 7 Centro de Investigación en Ciencias de la Salud (CICSA), Facultad de Ciencias de la Salud, Universidad Anáhuac México Campus Norte, Naucalpan, Mexico

**Keywords:** Dyspnea, Forced expiratory volume at first second, Peripheral oxygen saturation, Pulse oximetry, Inflammatory mediators, Cystic fibrosis, Glycine

## Abstract

**Background:**

Patients with cystic fibrosis (CF) have airway inflammation that contributes to symptoms and to pulmonary function derangement. Current drugs used to diminish airway inflammation improve the clinical and spirometric status of patients with CF, but their use is limited due to their undesired side effects, for example, glucose intolerance, growth retardation, and cataracts with corticosteroids, gastrointestinal toxicity with ibuprofen, and macrolide resistance with azythromycin. Glycine is known to decrease activation of inflammatory cells, including alveolar macrophages and neutrophils, and is relatively inexpensive, palatable, and virtually devoid of untoward effects. These features make glycine a good candidate for antiinflammatory treatment of CF. Thus, we aimed to explore whether glycine can exert a beneficial effect in a population of patients with CF.

**Methods:**

This was a randomized, double blinded, cross-over pilot clinical trial. Subjects with CF received, in random order, oral glycine (0.5 g/kg/day, dissolved in any liquid) and placebo (glass sugar), each during 8 weeks with an intermediate 2-week wash-out period.

**Results:**

Thirteen subjects aged 6–23 years, 8 females, completed the two arms of the study. As compared with placebo, after glycine intake patients had better symptom questionnaire scores (*p* = 0.02), mainly regarding sputum features and dyspnea. While spirometric variables tended to decline during placebo intake, they remained stable or even increased during glycine treatment (*p* = 0.04 to *p* = 0.003). In this context, FEV_1_ declined 8.6% after placebo and increased 9.7% at the end of the glycine period. Pulse oximetry improved after glycine intake (*p* = 0.04 vs. placebo). TNF-α in serum and IL-6 and G-CSF in sputum tended to decline at the end of the glycine period (*p* = 0.061, *p* = 0.068 and *p* = 0.04, respectively, vs placebo). Glycine was remarkably well tolerated.

**Conclusions:**

The clinical, spirometric and inflammatory status of subjects with CF improved after just 8 weeks of glycine intake, suggesting that this amino acid might constitute a novel therapeutic tool for these patients. Thus, further studies are warranted.

**Trial registration:**

www.clinicaltrials.gov, registration number: NCT01417481, date of registration: March 12, 2012.

**Electronic supplementary material:**

The online version of this article (10.1186/s12890-017-0528-x) contains supplementary material, which is available to authorized users.

## Background

Cystic fibrosis (CF) is a multisystemic disease caused by a mutation in the gene that codifies for the CF transmembrane conductance regulator (CFTR), a chloride channel located in the apical membrane of epithelial cells from several tissues. In the airways, this CFTR abnormality provokes depletion of the periciliary liquid, which alters ciliary movement and decreases efficacy of the mucociliary escalator. In addition, the loss of CFTR-mediated bicarbonate secretion causes acidification of the airway surface liquid, favoring the impairment of airway host defense mechanisms [1]. These defects render airway mucus thick and dehydrated. Neutrophils and proinflammatory cytokines such as interleukin (IL)-8 can be retrieved from the bronchoalveolar lavage of children with CF, even if bacterial infection has never occurred, which suggests that an aseptic inflammatory process is present from the early years of life [2, 3]. All of these mechanisms favor the development of recurrent infections that further stimulate the inflammatory process [4].

Treatment of subjects with cystic fibrosis (CF) is mainly directed toward nutritional improvement, airway mucus fluidification and control of exacerbations. However, a further therapeutic approach is to attempt to diminish airway inflammation, and corticosteroids, high-dose ibuprofen, and azithromycin have been used for this purpose [5, 6]. Although clear evidences exist that these drugs partially avoid pulmonary function derangement in patients with CF, their use is very limited due to their undesired side effects [6–9], such as glucose intolerance, diabetes, growth retardation, and cataracts with corticosteroids, gastrointestinal toxicity with ibuprofen, and macrolide resistance with azythromycin. It has been estimated that in the United States only 5% and 8% of subjects with CF use oral corticosteroids or high-dose ibuprofen, respectively [10]. Therefore, there is a need for therapeutic alternatives aimed at controlling airway inflammation without serious toxic effects.

Glycine is a nonessential amino acid that, in addition to its constitutive role in proteins, is also an agonist for the so-called glycine receptors (GlyR). GlyR are chloride channels that upon activation, promote entry of chloride ions into the cell, favoring hyperpolarization of the plasma membrane [11]. In neurons, glycine functions as an inhibitory neurotransmitter, while in the liver-resident population of macrophages (Kupffer cells), alveolar macrophages and neutrophils this amino acid lowers their sensitivity to proinflammatory stimuli [12]. In 1996, Ikejima et al. [13] demonstrated that a diet enriched with 5% glycine diminished liver lesions and mortality in a rat model of endotoxic shock and prevented the rise of serum tumor necrosis factor alpha (TNF-α). In 2000, Wheeler et al. [14] observed in this same model that 4-week glycine administration also decreased neutrophilic pulmonary infiltration. Studies published in 2008 by Alarcón-Aguilar et al. [15] and in 2010 by Almanza-Pérez et al. [16] found that glycine diminished the expression and serum levels of TNF-α and IL-6 in mouse adipose tissue. In vitro studies have corroborated that glycine inhibits the production of TNF-α and superoxide anion in lipopolysaccharide-stimulated alveolar macrophages [17], as well as of TNF-α and IL-6 in a pre-adipocyte (3 T3-L1) cell line [18]. This latter effect appears to be mediated by interference with activation of nuclear factor kappa B (NFκB) [19]. In humans, oral glycine has been already employed for treatment of some diseases, apparently without side effects [20–22]. Commercial glycine is a water-soluble, crystalline white powder with a moderately sweet flavor and is relatively inexpensive (about $20 US dollars per kg in Mexico). Due to all of the previously mentioned features, glycine is currently considered an anti-inflammatory substance, but up to our knowledge its use in subjects with CF has never been explored. Thus, in the present pilot study we hypothesized that glycine will improve the clinical, spirometric and inflammatory status in these patients.

## Methods

In this randomized, double blinded, cross-over clinical trial, ambulatory subjects were recruited if they fulfilled the following criteria: 1) A diagnosis of CF, established by at least one phenotypic feature of CF and demonstration of a dysfunctional CFTR through sweat chloride >60 mmol/L in two separate samples or identification of a CFTR mutation in both alleles; 2) Over 6 years of age, so they can be able to cooperate in the spirometric evaluation; 3) Without modification of their CF treatment in the last 30 days, and 4) Free of exacerbations in the last 30 days and without acute respiratory disease in the last 15 days. Subjects were excluded if they had participated in another research protocol including those involving anti-inflammatory or antimicrobial agents in the last 3 months.

Before entering into the study, an informed consent letter was signed out by adult patients or by the parents or guardians of pediatric patients, and an assent letter was also signed out by children aged 7 years or over. During the study, subjects underwent six evaluations at weeks 0, 4, 8, 10, 14, and 18. At the initial visit (week 0), patients were randomly allocated to the experimental (0.5 g/kg/day glycine) or placebo (same amount of glass sugar) treatment arms. We chose glass sugar as placebo because its appearance, white color, sweet taste, solubility in liquids, and texture closely resembles that of glycine. Allocation was done by using a computer-generated list of random numbers. There was no specific stratification by sex, age or any other variable before random allocation. Selection of the glycine dose was based in previous clinical reports that used from ~0.25 to ~1 g/kg/day [20, 23, 24]. No attempts were made to homogenize the usual treatment, so patients continued all other standard therapy as prescribed by their treating physicians. Due to the cross-over nature of our study, this last approach allowed us to evaluate any effect of glycine into the context of the habitual management.

After the first 8 weeks of treatment, glycine or placebo were discontinued during 2 weeks (this wash-out period was considered sufficient to allow complete glycine clearance, taking into account its short half-life of about 26–245 min) [25], and during the next 8-week period the subject was switched to the other treatment.

At each of the six visits, subjects were evaluated by means of the following: 1) A questionnaire applied to the patients and/or their parents inquiring about evolution of symptoms in the last 2 weeks with respect to: a) frequency of cough, b) features of sputum, i.e., amount and purulence, c) appetite, d) degree of dyspnea, and e) energy level. Each item was answered in Liker-type scale ranging from 1 (better) to 5 (worse); 2) Recording of subjects’ weight and height (scale/stadiometer model 420, Bame, México), as well as heart rate and respiratory rate by physical examination; 3) Measurement of SpO_2_; 4) Spirometric evaluation; 5) A blood sample obtained from a peripheral vein for hemogram and serum biomarkers determination; 6) A sputum sample spontaneously expectorated for biomarker determination. At each visit, the patients received a 500 g supply of glycine or placebo, respectively, both packaged in identical plastic bottles to assure that participants and/or their parents ignore the nature of the content. Patients and/or their parents were instructed to administer the supplement three times a day dissolved in any liquid (the corresponding amount to take in each dose was clearly marked in a 10-ml cylindrical plastic container). There were no special instructions about taking the supplement at a specific hour of the day or in relation with meals. Potential untoward effects of the treatments were inquired about at all subsequent visits.

### Spirometry

A more complete description of spirometry, pulse oximetry and biochemical determinations can be found in the online-only Additional file 1.

Spirometric evaluation was carried out according to international guidelines [26], and forced vital capacity (FVC), forced expiratory volume at first second (FEV_1_), FEV_1_/FVC ratio, forced expiratory flows at 25%, 50% and 75% of the FVC (FEF_25_, FEF_50_, FEF_75_, respectively), and maximal forced expiratory flow (FEFmax) were derived and expressed as percentage of predicted values [27].

### Pulse oximetry

SpO_2_ was measured through a pulse oximeter positioned in one of the participant’s fingertips.

### Biochemical determinations

Myeloperoxidase (MPO) was measured in serum and sputum supernatant by ELISA. Cytokine concentrations in serum and sputum supernatant were measured with magnetic bead multiplex immunoassays, including 17 analytes (IL-1β, IL-2, IL-4, IL-5, IL-6, IL-7, IL-8, IL-10, IL-12 (p70), IL-13, IL-17A, GCSF, GM-CSF, IFN-γ, MCP-1, MIP-1β, TNF-α). Serum glycine concentration was determined by using a commercial competitive ELISA kit.

### Data analysis

Baseline values were expressed as mean ± standard deviation, except for the biomarker concentrations that were expressed as median and range.

All changes observed at weeks 4 and 8 of the experimental and placebo arms were expressed as percentages of their respective baseline values, except for serum glycine determinations that were expressed in actual values (μg/ml). For clinical and spirometric variables, these percentages followed the normal distribution (Kolmogorov-Smirnov test), while for biomarkers they were log-transformed to reach normality. Due to the cross-over nature of the study, paired Student’s t-test was used to compare both arms regarding clinical and spirometric variables. Because blood and sputum samples could not be obtained at some visits, non-paired Student’s t-test was used to compare hemograms, serum and sputum biomarkers, and serum glycine. Concentration of biomarkers or glycine below the lower limit of detection received a value corresponding to one centesimal less than the minimum detectable value for that analyte. Nevertheless, some cytokines (IL-2, IL-5, IL-10, IL-17, and GM-CSF) were undetectable in more than 60% of serum samples, and they were excluded from the analysis. Finally, chi-square test, Pearson correlation coefficient, and straight line and curvilinear regressions were also used. Statistical significance was set at one-tailed *p* < 0.05.

## Results

**Fig. 1:**
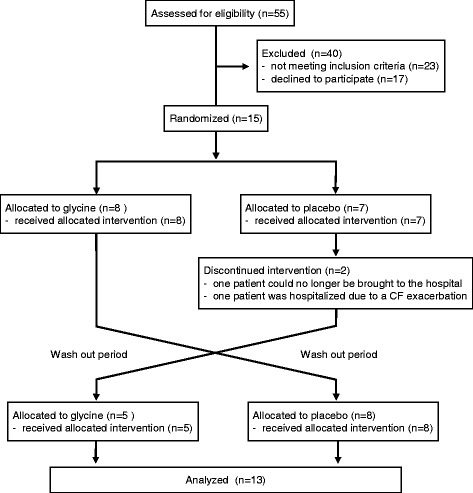
Flow diagram describing the selection process and follow up of participants

The recruitment and follow-up of participants are detailed in Fig. 1. From the 15 subjects originally recruited, two were eliminated from the study after starting the placebo arm (one boy because his mother underwent a programmed surgery and he could no longer be brought to the hospital, and one girl because she was hospitalized for 2 weeks due to an exacerbation of CF). Thus, the final population comprised 13 subjects (eight females) aged 13.0 ± 5.4 years (mean ± standard deviation) whose general features at enrollment are depicted in Table 1 and online-only Additional file 2: Table S1.

**Table 1:** Baseline characteristics of patients with cystic fibrosis at their enrollment in the study and at the start of the placebo or glycine periods

		Value at the start of		
Variable	Value at enrollment	Placebo	Glycine	p*
Gender *(male*: *female)*	5:8	–	–	–
Age *(years)*	13.0 ± 5.4	–	–	–
BMI *(kg/m* ^*2*^ *)*	15.9 ± 1.5	16.1 ± 1.6	15.8 ± 1.5	0.32
Respiratory rate *(breaths/min)*	25.3 ± 4.8	22.8 ± 2.6	26.5 ± 5.0	**0.015**
Questionnaire score^a^	11.1 ± 3.6	10.1 ± 3.1	11.1 ± 3.1	0.21
SpO_2_ *(%)*	89.5 ± 7.5	92.8 ± 2.8	88.4 ± 7.6	**0.032**
FVC *(% of predicted)*	79.1 ± 30.8	84.0 ± 25.2	79.5 ± 28.0	0.34
FEV_1_ *(% of predicted)*	65.3 ± 34.4	66.8 ± 30.6	58.7 ± 27.9	0.25
FEV_1_/FVC *(% of predicted)*	80.4 ± 16.6	79.8 ± 14.7	74.1 ± 14.0	0.17
Serum glycine (μg/ml)	121.9 ± 23.3	131.8 ± 16.4	153.7 ± 35.1	0.29

Data correspond to frequency (gender) or mean ± standard deviation (*n* = 13)

Bold font was used to highlight statistically significant differences

^*^Statistical significance comparing Placebo vs Glycine (Student’s t-test or Mann-Whitney U test)

^a^Each of five items (cough, sputum, appetite, dyspnea, and energy) was answered in a 5-options Likert scale, ranging from 1 (better) to 5 (worse), for a possible total score value of 5–25

*BMI* body mass index, *CF* cystic fibrosis, *FEV*_*1*_ forced expiratory volume at first second, *FVC* forced vital capacity

At the beginning of the study, 11 (84.6%) subjects had airway colonization in their most recent evaluation, all by *Pseudomonas aeruginosa*, except for one by *Staphylococcus aureus*. All subjects were under treatment with pancreatic enzymes and nebulized salbutamol, while 12 received vitamin E, 8 inhaled dornase alpha, 5 oral or intramuscular multivitamins, 4 azithromycin, 2 inhaled tobramycin, 2 ibuprofen, and 2 inhaled hypertonic saline. All subjects had some degree of nail clubbing. During the study, 7 children initiated ciprofloxacin with or without other antibiotics because of exacerbation of CF symptoms (4 while in placebo, and 3 while in glycine).

Serum glycine concentrations at enrollment averaged 121.9 ± 23.3 μg/ml (Table 1). At weeks 4 and 8, glycine concentrations tended to be higher in the group receiving this amino acid, although differences with the control group lacked statistical significance (173.2 ± 39.8 vs placebo 134.8 ± 23.9 μg/ml, *p* = 0.21, and 217.7 ± 40.6 vs placebo 141.4 ± 35.6, *p* = 0.09, respectively; Additional file 3: Figure S1).

Both treatment arms were well tolerated, with none of the subjects reporting adverse effects potentially attributable to glycine or placebo. Adherence to treatment was inquired at each subsequent visit, and it was catalogued as good by the majority of patients and parents.

### Effect of glycine on clinical and spirometric status

Baseline values of clinical and spirometric variables were almost similar in both treatment arms, excepting for a higher respiratory rate and a lower SpO_2_ at the beginning of the glycine period (Table 1 and online-only Additional file 2: Table S1).

**Fig. 2:**
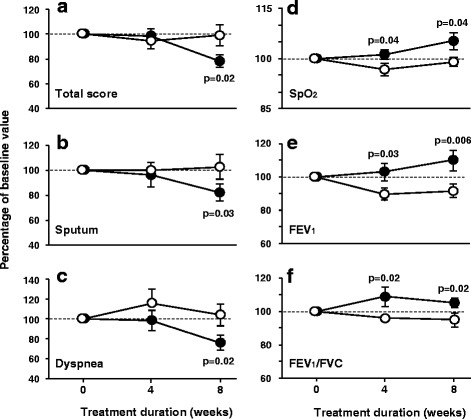
Changes in symptom scores, main spirometric variables and pulse oximetry in subjects with cystic fibrosis during glycine and placebo intake. All data are expressed as percentage of their respective baseline values. Symbols correspond to mean ± standard error of 13 patients who received 0.5 g/kg/day glycine (filled circles) and placebo (empty circles) during 8 weeks in random order. Statistical significance was assessed through paired Student’s t-test. The symptoms total score, sputum features and dyspnea perception were assessed by a Liker-type scale ranging from 1 (better) to 5 (worse). SpO_2_ = peripheral blood oxygen saturation; FEV_1_ = forced expiratory volume at the first second; FVC = forced vital capacity

As can be seen in Table 2, after 8 weeks of treatment symptomatic improvement occurred with glycine administration, as compared with placebo, reaching statistically significant difference in the questionnaire total score (*p* = 0.02), mainly due to improvement in sputum features (*p* = 0.03) and dyspnea perception (*p* = 0.02) (Fig. 2a, b and c). Respiratory rate tended to be lower during glycine administration, as compared with the placebo period, reaching statistical significance at week 4 (*p* = 0.02). Notably, pulse oximetry showed higher SpO_2_ levels at weeks 4 and 8 after glycine ingestion, with *p* = 0.04 at either visit (Fig. 2d). Hemogram did not differ in both treatment arms.

**Table 2:** Changes of clinical data, spirometric variables, and biomarkers in patients with cystic fibrosis after 4 and 8 weeks of glycine or placebo treatment, expressed as percentage of their respective baseline values

	Placebo		Glycine		Comparison between groups^a^	
Variable	Week 4	Week 8	Week 4	Week 8	Week 4	Week 8
Weight	102.7 ± 2.8	103.6 ± 3.6	100.5 ± 3.9	101.6 ± 3.9	0.08	0.13
Height	100.1 ± 0.5	100.5 ± 0.6	100.2 ± 0.4	100.5 ± 0.5	0.28	0.49
Heart rate	109.4 ± 11.9	98.1 ± 10.8	106.2 ± 18.6	103.5 ± 21.7	0.31	0.24
Respiratory rate	116.0 ± 17.1	109.0 ± 23.0	101.4 ± 17.8	94.8 ± 20.0	**0.02**	0.06
Temperature	100.2 ± 1.6	100.1 ± 1.7	100.4 ± 1.7	100.0 ± 1.0	0.37	0.45
Questionnaire^b^						
Cough	94.9 ± 41.6	89.1 ± 17.8	100.6 ± 35.4	81.1 ± 45.3	0.35	0.25
Sputum	100.0 ± 22.3	102.6 ± 36.4	96.4 ± 35.7	82.0 ± 25.4	0.36	**0.03**
Appetite	98.7 ± 39.4	132.1 ± 91.4	94.9 ± 31.5	89.1 ± 39.6	0.39	0.08
Dyspnea	115.4 ± 52.0	103.8 ± 38.6	98.2 ± 35.6	75.6 ± 27.1	0.19	**0.02**
Energy	100.0 ± 73.6	111.5 ± 58.3	114.1 ± 59.3	84.6 ± 26.8	0.33	0.11
Total score	94.5 ± 23.8	98.7 ± 30.1	97.8 ± 22.1	77.7 ± 18.8	0.39	**0.02**
SpO_2_	96.8 ± 6.7	98.9 ± 5.1	101.1 ± 5.5	105.2 ± 9.0	**0.04**	**0.04**
Hemogram						
Hemoglobin	101.6 ± 5.8	98.6 ± 5.5	96.0 ± 12.4	98.5 ± 7.5	0.15	0.48
Leukocytes	116.0 ± 17.8	103.9 ± 23.2	101.1 ± 41.7	100.5 ± 43.9	0.20	0.42
Neutrophils	121.7 ± 28.9	107.0 ± 36.0	107.9 ± 76.4	103.5 ± 66.9	0.33	0.45
Platelets	98.1 ± 20.4	94.9 ± 17.0	92.3 ± 23.6	92.5 ± 17.3	0.31	0.39
Spirometry						
FVC	93.2 ± 12.9	100.6 ± 35.3	96.3 ± 19.7	104.1 ± 14.8	0.33	0.38
FEV_1_	89.7 ± 13.0	91.4 ± 14.6	102.9 ± 19.3	109.7 ± 22.9	**0.03**	**0.006**
FEV_1_/FVC	95.9 ± 3.1	94.9 ± 15.1	108.8 ± 20.4	105.2 ± 10.6	**0.02**	**0.02**
Serum biomarkers						
MPO	81.1 ± 454.5	51.2 ± 581.8	154.3 ± 465.4	36.6 ± 496.1	0.15	0.35
IL-1	156.3 ± 335.6	92.2 ± 404.2	86.4 ± 398.8	68.6 ± 420.6	**0.035**	0.26
IL-2^c^	–	–	–	–	–	–
IL-4	199.1 ± 428.7	140.3 ± 500.5	70.2 ± 467.2	197.9 ± 416.0	**0.042**	0.29
IL-5^c^	–	–	–	–	–	–
IL-6	409.4 ± 407.8	168.1 ± 460.9	275.3 ± 453.7	102.0 ± 482.6	0.23	0.22
IL-7	142.6 ± 393.2	120.7 ± 376.5	90.9 ± 382.4	108.5 ± 345.5	0.12	0.35
IL-8	101.0 ± 411.0	58.0 ± 450.0	188.6 ± 480.7	71.4 ± 596.0	0.14	0.40
IL-10^c^‡	–	–	–	–	–	–
IL-12	284.7 ± 553.7	182.1 ± 488.4	114.0 ± 560.9	209.1 ± 418.8	0.16	0.41
IL-13	206.8 ± 475.4	156.8 ± 459.3	55.8 ± 465.7	87.9 ± 442.1	**0.028**	0.16
IL-17 ‡	–	–	–	–	–	–
G-CSF	262.9 ± 436.8	168.7 ± 473.6	188.8 ± 421.2	83.6 ± 429.1	0.26	0.12
GM-CSF^c^	–	–	–	–	–	–
IFN-γ	276.3 ± 481.1	231.1 ± 534.1	112.0 ± 393.9	212.4 ± 450.0	0.058	0.45
MCP-1	144.9 ± 373.0	111.5 ± 368.5	115.6 ± 376.0	82.5 ± 381.3	0.24	0.18
MIP-1β	157.8 ± 399.4	86.9 ± 374.5	143.6 ± 412.4	107.9 ± 416.6	0.41	0.30
TNF-α	460.6 ± 458.3	159.8 ± 519.4	162.0 ± 736.1	40.7 ± 564.3	0.15	0.061
Sputum biomarkers						
MPO	99.5 ± 457.7	116.7 ± 470.4	229.1 ± 571.8	134.7 ± 498.3	0.11	0.41
IL-1	275.4 ± 490.8	97.7 ± 450.4	160.7 ± 492.4	80.9 ± 428.9	0.17	0.35
IL-2	155.8 ± 411.7	93.9 ± 441.6	93.0 ± 436.3	105.5 ± 363.0	0.077	0.38
IL-4	138.7 ± 377.4	112.8 ± 387.1	117.1 ± 415.4	96.4 ± 335.2	0.26	0.22
IL-5	107.9 ± 499.7	135.0 ± 509.5	108.1 ± 618.7	177.7 ± 459.8	0.50	0.33
IL-6	147.2 ± 398.2	149.3 ± 402.3	77.0 ± 435.1	100.0 ± 350.6	**0.031**	0.068
IL-7	104.3 ± 395.0	137.6 ± 420.2	70.4 ± 410.1	115.1 ± 365.0	0.083	0.30
IL-8	97.1 ± 372.6	113.3 ± 411.3	136.8 ± 491.7	82.7 ± 510.8	0.21	0.28
IL-10	115.6 ± 361.3	101.7 ± 403.3	92.7 ± 425.1	113.5 ± 364.6	0.20	0.35
IL-12	118.3 ± 421.0	116.9 ± 442.2	82.4 ± 408.3	147.0 ± 418.2	0.13	0.30
IL-13	135.6 ± 445.1	124.6 ± 422.3	66.5 ± 445.4	145.5 ± 412.1	0.051	0.35
IL-17	137.3 ± 364.4	130.0 ± 400.8	121.0 ± 417.1	117.0 ± 329.8	0.30	0.32
G-CSF	135.7 ± 396.7	146.8 ± 419.6	89.5 ± 413.9	82.8 ± 359.9	0.079	**0.040**
GM-CSF	116.9 ± 342.9	82.7 ± 369.4	85.1 ± 376.5	88.3 ± 324.3	**0.007**	0.32
IFN-γ	139.6 ± 386.6	116.1 ± 392.3	125.8 ± 436.2	105.9 ± 348.6	0.37	0.35
MCP-1	96.5 ± 348.6	182.3 ± 528.6	118.4 ± 448.2	101.0 ± 352.0	0.25	0.14
MIP-1β	89.1 ± 359.3	125.2 ± 437.9	152.2 ± 447.9	93.3 ± 398.6	**0.047**	0.24
TNF-α	133.0 ± 408.1	143.5 ± 457.1	180.3 ± 547.5	109.9 ± 385.7	0.29	0.27

Data correspond to arithmetic (clinical and spirometric variables) or geometric (serum and sputum biomarkers) mean ± standard deviation of the percentage of baseline

Bold font was used to highlight statistically significant differences

^a^ *p* from unpaired (for serum and sputum biomarkers, *n* = 9–12 per group) or paired (for the remaining variables, *n* = 13) Student’s *t*-test comparing Glycine vs Placebo periods

^b^Each item was originally answered in a 5-options Likert scale, ranging from 1 (better) to 5 (worse)

^c^Eliminated from the analysis due to the large amount of samples (>60%) below the lower limit of detection

*FEV*_*1*_ forced expiratory volume at first second, *FVC* forced vital capacity, *G-CSF* granulocyte colony stimulating factor, *GM-CSF* granulocyte/macrophage colony stimulating factor, *IFN-γ* interferon gamma, *IL* interleukin, *MCP-1* monocyte chemotactic protein 1, *MIP-1β* macrophage inflammatory protein 1β, *MPO* myeloperoxidase, *SpO*_*2*_ peripheral blood oxygen saturation, *TNF-α* tumor necrosis factor alpha

Previous studies have shown that respiratory function in subjects with CF usually declines over time [28–30] and, in agreement with this, in the present study spirometric variables tended to decline during the placebo period (Table 2 and Fig. 2e-f). In contrast, during glycine administration, respiratory function was stable or even increased with respect to the baseline value. Thus, comparison between placebo and glycine yielded statistically significant differences in the majority of spirometric variables, with *p* values ranging from 0.04 to 0.003. For example, at the end of the placebo period FEV_1_, the spirometric variable commonly employed to evaluate progression of the disease, declined to 91.4% ± 14.6% from baseline, *p* = 0.03, but increased up to 109.7% ± 22.9% from baseline after the glycine period, *p* = 0.006. Thus, this variable increased up to 9.7% while in glycine and declined 8.6% with placebo. To exemplify how raw changes occurred during the study, Additional file 4: Figure S2 illustrates dyspnea score, FEV_1_ and SpO_2_ modifications as they took place throughout the study.

### Effect of glycine on serum and sputum biomarkers

Biological samples could not be obtained at all visits because parents of 3 children did not give their consent for blood extraction, a 6-years old girl was unable to expectorate, and at some visits children refused the venous puncture (6 visits) or did not expectorate (6 visits).

Baseline values of serum and sputum biomarkers were not different in both treatment arms, although concentrations of serum TNF-α and sputum IL-6 tended to be higher at the beginning of the glycine period (Additional file 2: Table S1).

**Fig. 3:**
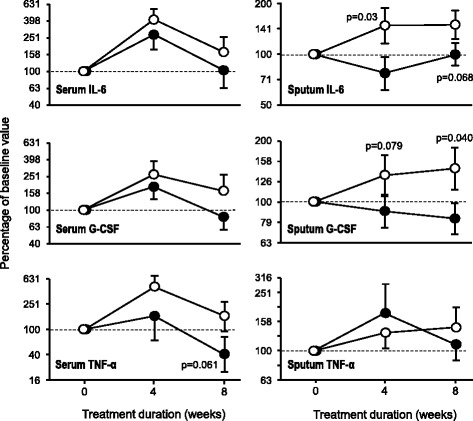
Changes in selected serum and sputum cytokines in subjects with cystic fibrosis during glycine and placebo intake. All data are expressed as percentage of their respective baseline values. Symbols correspond to mean ± standard error of 9–12 subjects who received 0.5 g/kg/day glycine (filled circles) and placebo (empty circles) during 8 weeks in random order. Statistical significance was assessed through non-paired Student’s t-test. G-CSF = granulocyte colony stimulating factor; IL-6 = interleukin 6; TNF-α = tumor necrosis factor alpha

Modifications of biomarkers at the end of each treatment arm are shown in Table 2. The only biomarker that reached statistically significant difference was the cytokine G-CSF in sputum, which was lower after glycine than after placebo (*p* = 0.040). Serum TNF-α and sputum IL-6 also displayed the same downward trend, although they did not attain statistical significance (*p* = 0.061 and *p* = 0.068, respectively). Changes in these cytokines are illustrated in Fig. 3. The remainder biomarkers were not different at the end of both treatment arms.

The Additional file 5: Figure S3 show raw changes in concentration of selected biomarkers as they occurred during the entire study in subjects who first took glycine and then placebo.

### Correlation between selected variables

**Fig. 4:**
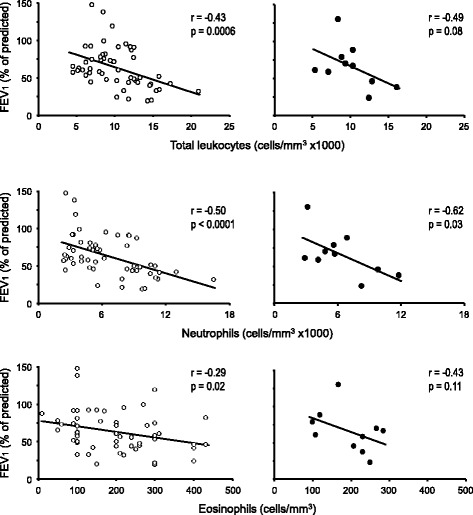
Correlations between peripheral blood leukocytes and FEV_1_ in subjects with cystic fibrosis. The scatter plots correspond either to all measures obtained at all visits (empty circles) or to the mean of the six visits for each patient (filled circles). The Pearson’s correlation coefficient (r) and its corresponding *p* value are shown in each panel

**Fig. 5:**
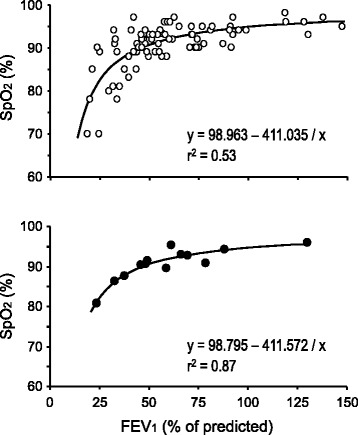
Correlation between forced expiratory volume at first second (FEV_1_) and peripheral oxygen saturation (SpO_2_) in subjects with cystic fibrosis. The scatter plots correspond either to all measures obtained at all visits (empty circles) or to the mean of the six visits for each patient (filled circles). The hyperbolic function formula and its associated coefficient of determination (r^2^) are shown in each panel

**Fig. 6:**
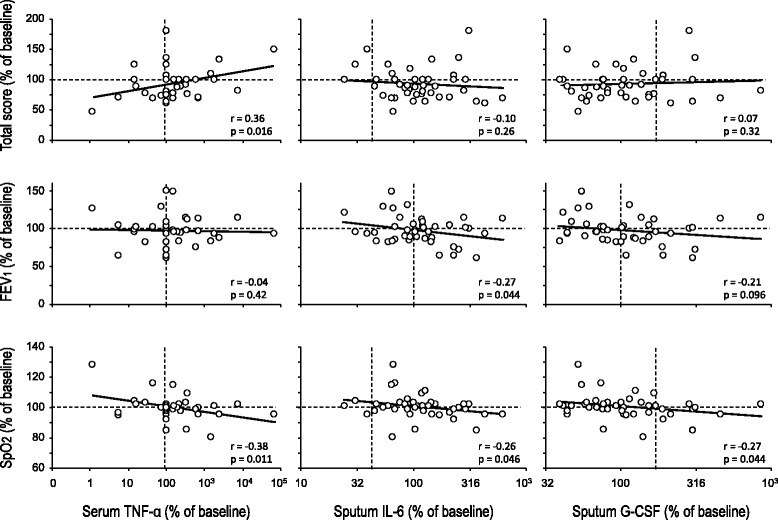
Relationship between selected cytokines and symptoms questionnaire, forced expiratory volume at first second (FEV_1_) and peripheral oxygen saturation (SpO_2_) in subjects with cystic fibrosis. Data correspond to all values observed at weeks 4 and 8, expressed as percentage of their respective baseline value at the beginning of the glycine or placebo period (week 0)

The number of total leukocytes, neutrophils and eosinophils had an inverse correlation with the percentage of predicted FEV_1_ (Fig. 4). Likewise, SpO_2_ values were progressively lower as FEV_1_ (% predicted) declined, but this fall was sharply accentuated when FEV_1_ fell beyond 40% of the predicted value (Fig. 5). Finally, Fig. 6 illustrates that during the entire study, increments of serum TNF-α, sputum IL-6 or sputum G-CSF were correlated with detrimental changes in the questionnaire total score, FEV_1_ and/or SpO_2_ levels.

## Discussion

In the present study, we found that daily oral intake of glycine was associated with symptomatic improvement and better respiratory function in subjects with CF, as compared with placebo. It has been estimated that subjects with CF lose about 2% of FEV_1_ annually [29, 30], and this decline has been considered a key marker of disease progression and a predictive factor for survival and health status [28]. In this context, one of the most encouraging findings of our study was that glycine was capable to restrain the FEV_1_ decline. In this sense, while FEV_1_ decreased by approximately 9% at the end of the 8-week period with placebo, the same subjects experienced an improvement of nearly 10% with glycine intake. Similarly, nearly all spirometric parameters tended to increase during glycine administration. Improvement in FEV_1_ was comparable to or even slightly better than changes reported during the usual anti-inflammatory treatment in subjects with CF, either with corticosteroids [31, 32], high-dose ibuprofen [33], or azithromycin [34]. It is reasonable to speculate that the mechanism by which glycine promoted better pulmonary function was due to its ability to inhibit the chemotaxis and activation of inflammatory cells, particularly neutrophils and macrophages [12–18]. Such an effect would be expected to reduce the thickening of the bronchial wall and to avoid the local release of proinflammatory mediators such as TNF-α, IL-1β and IL-6, which are known to promote excessive mucus production [35]. In line with this possibility, we found that glycine intake was associated with lower levels of sputum G-CSF, and a trend of lower serum TNF-α and sputum IL-6, and that changes of G-CSF and IL-6 tended to be inversely associated with changes in FEV_1_. This speculation is reinforced by the finding that the number of inflammatory cells (total leukocytes, neutrophils, and eosinophils) had a clear inverse association with pulmonary function as evaluated by FEV_1_. On the other hand, in 2011 Yim et al. [36] demonstrated for the first time that human airway smooth muscle expresses GlyR, and that activation of these receptors causes airway smooth muscle relaxation. Thus, in addition to its possible anti-inflammatory effect, glycine might have also improved pulmonary function through attenuation of bronchomotor tone.

We found that SpO_2_ levels progressively diminished as pulmonary function (FEV_1_) decreased, a finding that has been already described by other authors [37, 38]. Therefore, in our study the higher levels of SpO_2_ reached during glycine administration probably were reflecting the improvement of respiratory function. In this context, if glycine diminished bronchial inflammation and mucous plugging, better ventilation of previously hypoventilated alveolar regions would be expected to occur, thus leading to a more appropriate ventilation/perfusion (V′/Q’) relationship. A second possibility is that glycine was acting on the other element of the V′/Q’ relationship, i.e., the microcirculation. Although GlyR have not been demonstrated in vascular smooth muscle, it is known that these receptors are present in endothelial cells and cause hyperpolarization [39]. Because the endothelium is able to modulate the contraction of arteriolar muscle [40], it is also possible that glycine improved the blood perfusion of well ventilated zones, thus leading to a better V′/Q’ relationship.

Contrasting with the well-known untoward effects of corticosteroids, ibuprofen, or azithromycin, it was noteworthy that in our study, glycine was well tolerated, inasmuch as no patient presented symptoms attributable to the treatment.

Potential limitations of our study include the relatively small number of subjects, the relatively short time of follow up within each treatment arm, and a possible residual or carry over effect of glycine after the wash out period. Thus, larger and longer-term clinical trials, perhaps without a cross-over design, are needed to corroborate our results.

## Conclusion

In conclusion, the present study showed that daily administration of an oral supplement of 0.5 g/kg glycine during 8 weeks improved the clinical, spirometric and inflammatory status of subjects with CF, as compared with a placebo period. Thus, if replicated, our results suggest that glycine might constitute a novel therapeutic tool in patients with CF.

## Additional files

Additional file 1: A more complete description of spirometry, pulse oximetry, and biochemical determinations. (DOC 38 kb)

Additional file 2: Table S1. Additional Baseline Characteristics of Patients with Cystic Fibrosis at their Enrollment in the Study and at the Start of the Placebo or Glycine Periods. (DOC 124 kb)

Additional file 3: Figure S1. Changes in serum concentration of glycine in subjects with cystic fibrosis during glycine and placebo intake. Symbols correspond to mean ± standard error of *n* = 10 patients. (PDF 6 kb)

Additional file 4: Figure S2. Actual changes of dyspnea score, forced expiratory volume at first second (FEV_1_) and peripheral oxygen saturation (SpO_2_) in subjects with cystic fibrosis as occurred throughout the study. Randomly selected patients began the study (weeks 0–8) either with placebo (empty circles, *n* = 5) or glycine (filled circles, *n* = 8) and after a wash-out period these subjects were switched to the alternative treatment (weeks 10–18). All data are expressed as percentage of the baseline value at week 0. Symbols correspond to mean ± standard error. (PDF 115 kb)

Additional file 5: Figure S3. Actual changes of selected serum and sputum cytokines in the 8 subjects with cystic fibrosis who first received glycine (weeks 0–8) and then placebo (weeks 10–14). All data are expressed as percentage of the baseline value at week 0. G-CSF = granulocyte colony stimulating factor; IL-6 = interleukin 6; IL-8 = interleukin 8; MPO = myeloperoxydase; TNF-α = tumor necrosis factor alpha. (PDF 128 kb)

## Abbreviations

BMI: Body mass index
CF: Cystic fibrosis
CFTR: Cystic fibrosis transmembrane conductance regulator
FEF_25_: Forced expiratory flow at 25% of the vital capacity
FEF_50_: Forced expiratory flow at 50% of the vital capacity
FEF_75_: Forced expiratory flow at 75% of the vital capacity
FEFmax: Maximal forced expiratory flow
FEV_1_: Forced expiratory volume at the first second
FVC: Forced vital capacity
G-CSF: Granulocyte colony stimulating factor
GlyR: Glycine receptor
GM-CSF: Granulocyte/macrophage colony stimulating factor
IFN-γ: Interferon gamma
IL: Interleukin
MCP-1: Monocyte chemotactic protein 1
MIP-1β: Macrophage inflammatory protein 1β
MPO: Myeloperoxidase
NFκB: Nuclear factor kappa B
SpO_2_: Peripheral blood oxygen saturation
TNF-α: Tumor necrosis factor alpha
V′/Q’: Ventilation/perfusion ratio
