# Supplementary material for: Effect of oral glycine on the clinical, spirometric and inflammatory status in subjects with cystic fibrosis: a pilot randomized trial
Source: BMC Pulm Med. 2017 Dec 15;17:206. doi: 10.1186/s12890-017-0528-x (PMC5732413; doi:10.1186/s12890-017-0528-x)
Supplement: Supplementary file 1 — A more complete description of spirometry, pulse oximetry, and biochemical determinations. (DOC 38 kb) [file 12890_2017_528_MOESM1_ESM.doc]

**SUPPLEMENTARY METHODS**

**Effect of oral glycine on the clinical, spirometric and inflammatory status in subjects with cystic fibrosis. A pilot randomized trial**

Mario H. Vargas, Rosangela Del-Razo-Rodríguez, Amando López-García, José Luis Lezana-Fernández, Jaime Chávez, María E.Y. Furuya, and Juan Carlos Marín-Santana

**Methods**

**Spirometry**

Spirometric evaluation was carried out according to international guidelines [1]. Briefly, after the procedure was explained and exemplified, and with the subject in the sitting position and using a nose clip, a full inspiration was followed by a forceful expiration into the spirometer (Master Screen Body, Jaeger, CareFusion, Hoechberg, Germany, or Elite, Medical Graphics Corporation, St Paul, MN, USA). This maneuver was repeated up to eight times until the criteria of acceptability and repeatability was obtained for at least three maneuvers. From these, forced vital capacity (FVC), forced expiratory volume at first second (FEV_1_), FEV_1_/FVC ratio, forced expiratory flows at 25%, 50% and 75% of the FVC (FEF_25_, FEF_50_, FEF_75_, respectively), and maximal forced expiratory flow (FEFmax) were derived and expressed as percentage of predicted values [2].

**Pulse oximetry**

SpO_2_ was measured through a pulse oximeter (Life Scope 9, BSM-8302J, Nihon Kohden, Seoul, Korea, or Onyx II 9550, Nonin, Plymouth, MN, USA) positioned in one of the participant’s fingertips.

**Biochemical determinations**

A 2-ml blood sample was obtained from a peripheral vein and immediately refrigerated. Before 24 h the blood sample was centrifuged at 1800 rpm during 10 min and serum was aliquoted and stored at -70°C until analyzed. Spontaneously expectorated sputum samples were immediately frozen, and before 24 h they were thawed, weighted and treated with 0.1% dithiothreitol (Sputolysin Reagent, Calbiochem Corp, San Diego, CA, USA) at a 4:1 (v/w) ratio. This mixture was high-speed vortexed twice during 15-20 s at 37°C, and then diluted with phosphate buffer (4-fold the volume of dithiothreitol). All the fluid was filtered using a 40 μm nylon mesh (BD Falcon, NJ, USA) and centrifuged at 1800 rpm during 10 min at 4°C. The supernatant was carefully collected, aliquoted and stored at -70°C until analyzed.

Myeloperoxidase (MPO) was measured in serum and sputum supernatant by ELISA (Quantikine Human Immunoassay, R&D systems, Minneapolis, MN, USA). Cytokine concentrations in serum and sputum supernatant were measured with magnetic bead multiplex immunoassays (Bio-Plex ProTM Assay, Human Cytokine 17-Plex Panel, Bio-Rad Laboratories, USA). xMAP technology was used for simultaneous measurement of 17 analytes (IL-1β, IL-2, IL-4, IL-5, IL-6, IL-7, IL-8, IL-10, IL-12 (p70), IL-13, IL-17A, GCSF, GM-CSF, IFN-γ, MCP-1, MIP-1β, TNF-α). Briefly, 50 μl of non-diluted samples or standard were added to a 96-well assay plate containing coupled magnetic beads followed by incubation in the dark at room temperature with shaking at 300 rpm during 30 min. After washing, the antibody was added and incubated for 30 min. Afterward, streptavidin-phycoerythrin conjugated was added as fluorescent indicator. Data for the reactions from 96-well plate were obtained using a Bio-Plex 200 system and analyzed with the Bio-Plex Manager 6.0 software. Optimized standard curves were generated for each analyte and returned the reading data as median fluorescence intensity (MFI) and concentration (pg/ml).

Serum glycine concentration was determined by using a commercial competitive ELISA kit (LDN Labor Diagnostika Nord, Norhorn, Germany) with analytical sensitivity of 3.3 µg/ml.

**Abbreviations**

BMI, body mass index; CF, cystic fibrosis; CFTR, cystic fibrosis transmembrane conductance regulator; FEF_25_, forced expiratory flow at 25% of the vital capacity; FEF_50_, forced expiratory flow at 50% of the vital capacity; FEF_75_, forced expiratory flow at 75% of the vital capacity; FEFmax, maximal forced expiratory flow; FEV_1_, forced expiratory volume at the first second; FVC, forced vital capacity; G-CSF, granulocyte colony stimulating factor; GlyR, glycine receptor; GM-CSF, granulocyte/macrophage colony stimulating factor; IFN-γ, interferon gamma; IL, interleukin; MCP-1, monocyte chemotactic protein 1; MIP-1β, macrophage inflammatory protein 1β; MPO, myeloperoxidase; NFκB, nuclear factor kappa B; SpO_2_, peripheral blood oxygen saturation; TNF-α, tumor necrosis factor alpha; V'/Q', ventilation/perfusion ratio.

**References for Supplemental Methods**

1. Miller MR, Hankinson J, Brusasco V, Burgos F, Casaburi R, Coates A, Crapo R, Enright P, van der Grinten CP, Gustafsson P, et al: **Standardisation of spirometry.** *Eur Respir J* 2005, **26:**319-338.

2. Hankinson JL, Odencrantz JR, Fedan KB: **Spirometric reference values from a sample of the general U.S. population.** *Am J Respir Crit Care Med* 1999, **159:**179-187.
