# Supplementary material for: Effect of oral glycine on the clinical, spirometric and inflammatory status in subjects with cystic fibrosis: a pilot randomized trial
Source: BMC Pulm Med. 2017 Dec 15;17:206. doi: 10.1186/s12890-017-0528-x (PMC5732413; doi:10.1186/s12890-017-0528-x)
Supplement: Supplementary file 2 — Additional Baseline Characteristics of Patients with Cystic Fibrosis at their Enrollment in the Study and at the Start of the Placebo or Glycine Periods. (DOC 124 kb) [file 12890_2017_528_MOESM2_ESM.doc]

**SUPPLEMENTARY TABLE S1**

**Effect of oral glycine on the clinical, spirometric and inflammatory status in subjects with cystic fibrosis. A pilot randomized trial**

Mario H. Vargas, Rosangela Del-Razo-Rodríguez, Amando López-García, José Luis Lezana-Fernández, Jaime Chávez, María E.Y. Furuya, and Juan Carlos Marín-Santana

| Supplementary Table 1. Additional Baseline Characteristics of Patients with Cystic Fibrosis at their Enrollment in the Study and at the Start of the Placebo or Glycine Periods | | | | |
| --- | --- | --- | --- | --- |
|  |  | Value at the start of | |  |
| Variable | Value at enrollment | Placebo | Glycine | p* |
| Questionnaire scores † |  |  |  |  |
| Cough | 2.7 ± 0.9 | 2.6 ± 1.0 | 2.7 ± 0.8 | 0.42 |
| Sputum | 2.8 ± 1.3 | 2.6 ± 1.3 | 3.0 ± 1.0 | 0.20 |
| Appetite | 1.8 ± 0.7 | 1.5 ± 0.7 | 1.9 ± 0.6 | 0.072 |
| Dyspnea | 2.0 ± 1.2 | 1.6 ± 0.9 | 2.0 ± 1.2 | 0.18 |
| Energy | 1.8 ± 0.6 | 1.7 ± 0.5 | 1.8 ± 0.7 | 0.38 |
| Total score | 11.1 ± 3.6 | 10.1 ± 3.1 | 11.1 ± 3.1 | 0.21 |
| Hemogram |  |  |  |  |
| Hemoglobin *(g/dl)* | 15.2 ± 1.8 | 14.9 ± 1.1 | 15.4 ± 1.9 | 0.24 |
| Leukocytes *(/mm^3^)* | 9765 ± 3845 | 10145 ± 3908 | 10624 ± 4813 | 0.41 |
| Neutrophils *(/mm^3^)* | 5991 ± 3500 | 6538 ± 3684 | 6940 ± 4305 | 0.42 |
| Platelets *(/mm^3^)* | 331800 ± 121761 | 335750 ± 139790 | 329222 ± 85879 | 0.45 |
| Serum biomarkers |  |  |  |  |
| MPO *(ng/ml)* | 706.7 (184.8 - 6292.5) | 706.7 (169.0 - 2360.95) | 567.5 (209.5 - 6292.5) | 0.43 |
| IL-1 *(pg/ml)* | 0.36 (0.06 - 0.93) | 0.37 (0.03 - 0.63) | 0.56 (0.06 - 2.16) | 0.28 |
| IL-2 *(pg/ml)* ‡ | - - - | - - - | - - - | - - - |
| IL-4 *(pg/ml)* | 0.08 (0.01 - 0.37) | 0.11 (0.01 - 0.22) | 0.08 (0.01 - 0.37) | 0.50 |
| IL-5 *(pg/ml)* ‡ | - - - | - - - | - - - | - - - |
| IL-6 *(pg/ml)* | 2.47 (0.17 - 14.18) | 1.87 (0.17 - 14.56) | 2.47 (0.65 - 17.38) | 0.20 |
| IL-7 *(pg/ml)* | 2.66 (0.10 - 10.41) | 2.61 (0.10 - 6.32) | 2.66 (1.19 - 10.41) | 0.22 |
| IL-8 *(pg/ml)* | 4.79 (1.73 - 576.91) | 4.79 (1.80 - 123.76) | 5.54 (1.73 - 576.91) | 0.50 |
| IL-10 *(pg/ml)* ‡ | - - - | - - - | - - - | - - - |
| IL-12 *(pg/ml)* | 0.14 (0.13 - 4.13) | 0.13 (0.13 - 3.03) | 1.62 (0.13 - 14.3) | 0.12 |
| IL-13 *(pg/ml)* | 2.17 (0.10 - 4.20) | 2.12 (0.10 - 4.2) | 2.31 (0.10 - 5.76) | 0.20 |
| IL-17 *(pg/ml)* ‡ | - - - | - - - | - - - | - - - |
| G-CSF *(pg/ml)* | 1.85 (0.32 - 9.73) | 1.67 (0.09 - 3.73) | 1.85 (0.93 - 9.73) | 0.19 |
| GM-CSF *(pg/ml)* ‡ | - - - | - - - | - - - | - - - |
| IFN-γ *(pg/ml)* | 4.91 (0.23 - 35.00) | 4.91 (0.23 - 29.3) | 8.71 (0.24 – 35.00) | 0.31 |
| MCP-1 *(pg/ml)* | 19.06 (10.28 - 75.50) | 19.06 (5.87 - 35.89) | 19.73 (10.28 - 75.50) | 0.17 |
| MIP-1β *(pg/ml)* | 15.23 (4.99 - 73.01) | 20.94 (1.62 - 58.42) | 15.23 (3.24 - 73.01) | 0.43 |
| TNF-α *(pg/ml)* | 0.07 (0.01 - 0.87) | 0.01 (0.01 - 1.10) | 0.18 (0.01 - 0.87) | 0.052 |
| Sputum biomarkers |  |  |  |  |
| MPO *(ng/ml)* | 52547 (3842 - 175762) | 42130 (605 - 133486) | 56511 (135 - 175762) | 0.33 |
| IL-1 *(pg/ml)* | 1866 (152 - 15177) | 2079 (130 - 6105) | 1562 (232 - 15177) | 0.44 |
| IL-2 *(pg/ml)* | 5.54 (0.38 - 9.82) | 5.01 (0.38 - 10.69) | 4.63 (2.24 - 9.82) | 0.50 |
| IL-4 *(pg/ml)* | 1.91 (0.41 - 3.40) | 2.05 (0.29 - 2.63) | 1.89 (0.41 - 3.40) | 0.38 |
| IL-5 *(pg/ml)* | 0.27 (0.01 - 0.68) | 0.17 (0.01 - 0.48) | 0.25 (0.01 - 0.68) | 0.29 |
| IL-6 *(pg/ml)* | 7.2 (0.58 - 69.92) | 5.24 (0.58 - 7.59) | 6.97 (2.88 - 69.92) | 0.06 |
| IL-7 *(pg/ml)* | 1.34 (0.21 - 4.66) | 1.83 (0.21 - 2.76) | 1.65 (0.55 - 4.66) | 0.28 |
| IL-8 *(pg/ml)* | 3367 (765 - 18977) | 1059 (741 - 4641) | 1814 (765 - 18977) | 0.10 |
| IL-10 *(pg/ml)* | 1.4 (0.64 - 3.06) | 1.37 (0.64 - 1.52) | 1.36 (0.64 - 3.06) | 0.37 |
| IL-12 *(pg/ml)* | 4.71 (0.35 - 28.01) | 4.92 (0.35 - 8.62) | 4.92 (0.76 - 28.01) | 0.50 |
| IL-13 *(pg/ml)* | 1.28 (0.09 - 5.95) | 1.37 (0.09 - 2.96) | 1.52 (0.35 - 5.95) | 0.37 |
| IL-17 *(pg/ml)* | 20.29 (5.21 - 37.70) | 18.01 (2.56 - 29.91) | 19.81 (5.21 - 37.70) | 0.30 |
| G-CSF *(pg/ml)* | 53.26 (9.41 - 199.92) | 40.25 (9.41 - 84.70) | 49.51 (26.80 - 199.92) | 0.11 |
| GM-CSF *(pg/ml)* | 49.74 (42.82 - 67.32) | 49.53 (34.11 - 59.50) | 49.95 (42.82 - 67.73) | 0.22 |
| IFN-γ *(pg/ml)* | 79.32 (8.48 - 163.48) | 108.32 (15.19 - 146.90) | 88.76 (8.48 - 163.48) | 0.50 |
| MCP-1 *(pg/ml)* | 9.14 (6.17 - 16.89) | 9.33 (2.78 - 25.61) | 10.07 (6.17 - 16.89) | 0.28 |
| MIP-1β *(pg/ml)* | 14.7 (6.81 - 56.71) | 8.95 (4.76 - 24.15) | 14.58 (6.81 - 56.71) | 0.11 |
| TNF-α *(pg/ml)* | 97.96 (5.64 - 544.67) | 100.60 (2.50 - 188.63) | 99.83 (5.64 - 544.67) | 0.39 |
| Data correspond to mean ± standard deviation for clinical variables (n=13) and hemograms (n=8-9), and to median and range for serum (n=9) and sputum (n=11) biomarkers.  Bold font was used to highlight statistically significant differences.  * Statistical significance comparing Placebo vs Glycine (Student’s t-test or Mann-Whitney U test).  † Each item was answered in a 5-options Likert scale, ranging from 1 (better) to 5 (worse).  ‡ Eliminated from the analysis due to the large amount of serum samples (>60%) with concentration below the lower limit of detection.  G-CSF=granulocyte colony stimulating factor; GM-CSF=granulocyte/macrophage colony stimulating factor; IFN-γ=interferon gamma; IL=interleukin; MCP-1=monocyte chemotactic protein 1; MIP-1β=macrophage inflammatory protein 1β; MPO=myeloperoxidase; TNF-α=tumor necrosis factor alpha. | | | | |
