# Supplementary figures and images for: Effect of oral glycine on the clinical, spirometric and inflammatory status in subjects with cystic fibrosis: a pilot randomized trial
Source: BMC Pulm Med. 2017 Dec 15;17:206. doi: 10.1186/s12890-017-0528-x (PMC5732413; doi:10.1186/s12890-017-0528-x)

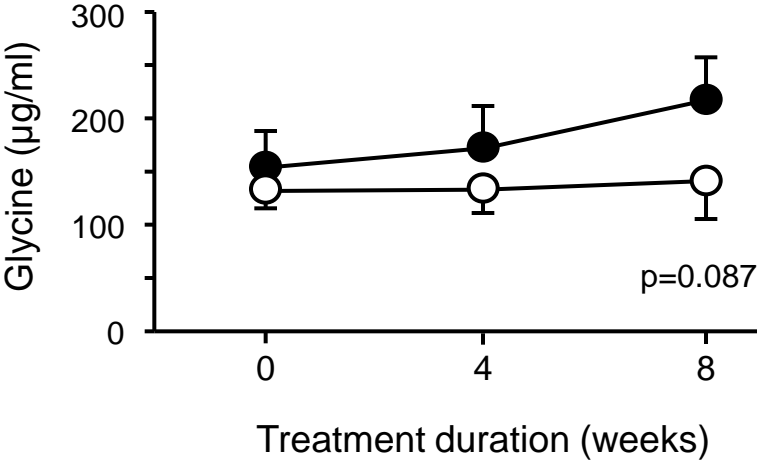

Supplement: Supplementary file 3 — Changes in serum concentration of glycine in subjects with cystic fibrosis during glycine and placebo intake. Symbols correspond to mean ± standard error of n = 10 patients. (PDF 6 kb) [file 12890_2017_528_MOESM3_ESM.pdf]

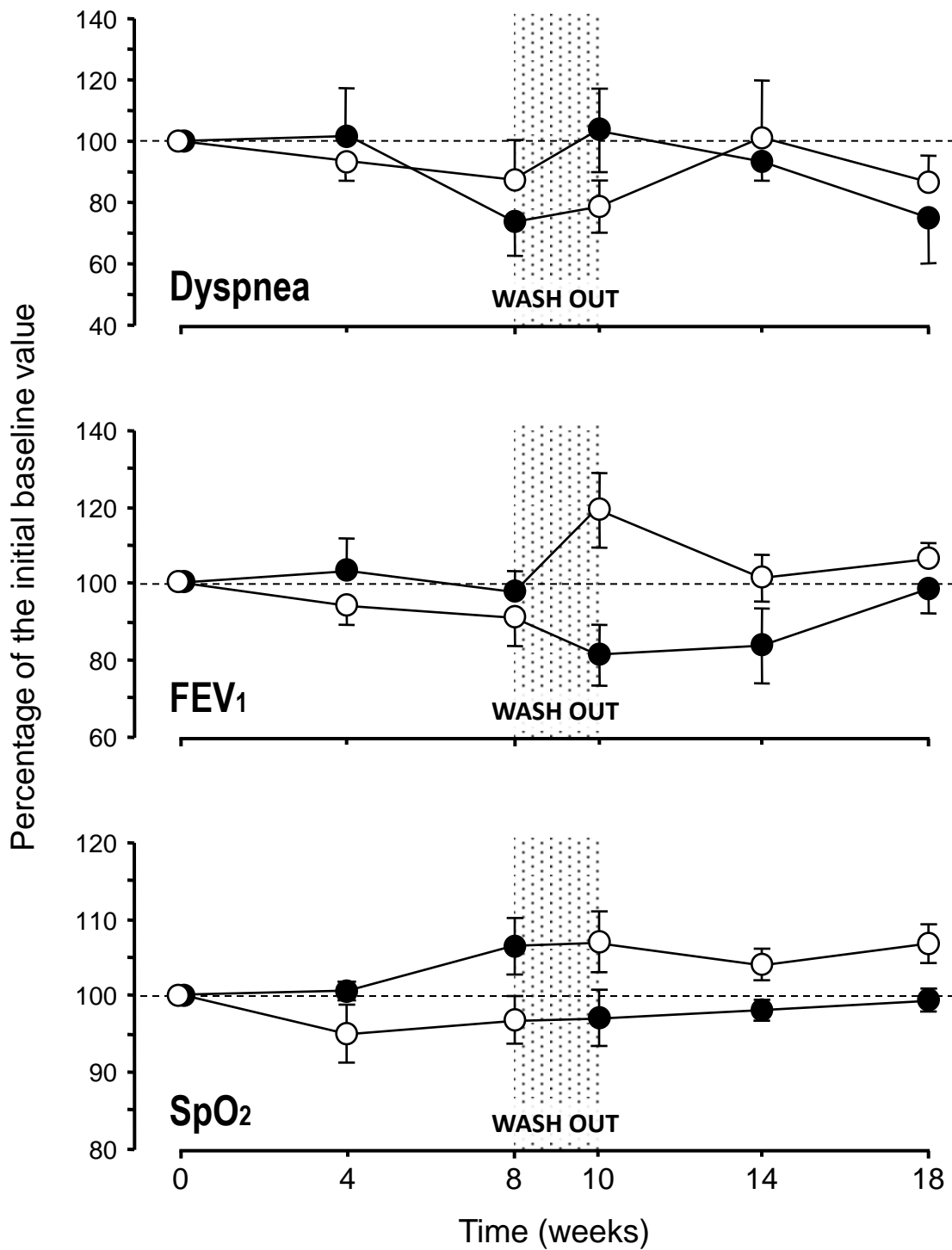

Supplement: Supplementary file 4 — Actual changes of dyspnea score, forced expiratory volume at first second (FEV1) and peripheral oxygen saturation (SpO2) in subjects with cystic fibrosis as occurred throughout the study. Randomly selected patients began the study (weeks 0–8) either with placebo (empty circles, n = 5) or glycine (filled circles, n = 8) and after a wash-out period these subjects were switched to the alternative treatment (weeks 10–18). All data are expressed as percentage of the baseline value at week 0. Symbols correspond to mean ± standard error. (PDF 115 kb) [file 12890_2017_528_MOESM4_ESM.pdf]

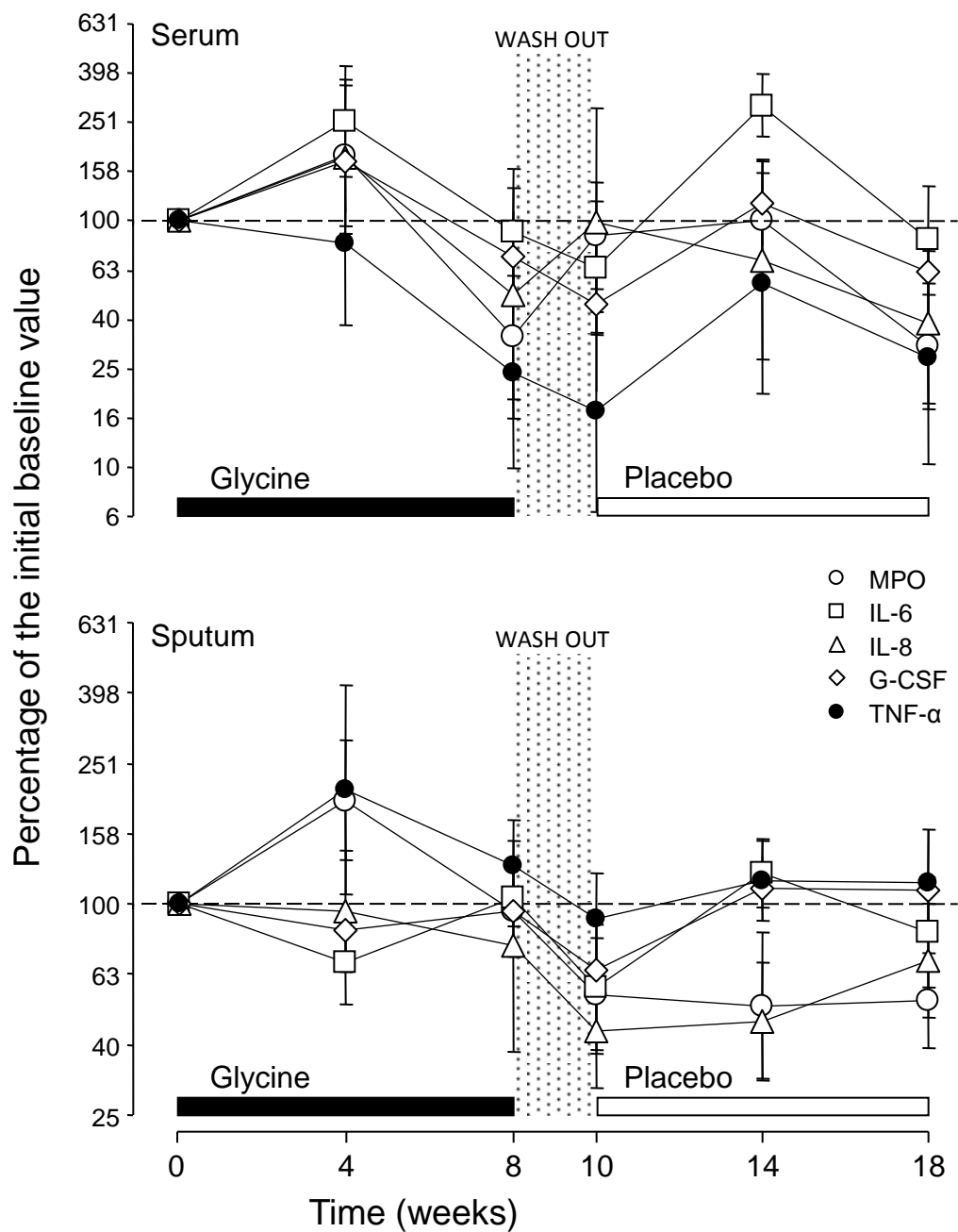

Supplement: Supplementary file 5 — Actual changes of selected serum and sputum cytokines in the 8 subjects with cystic fibrosis who first received glycine (weeks 0–8) and then placebo (weeks 10–14). All data are expressed as percentage of the baseline value at week 0. G-CSF = granulocyte colony stimulating factor; IL-6 = interleukin 6; IL-8 = interleukin 8; MPO = myeloperoxydase; TNF-α = tumor necrosis factor alpha. (PDF 128 kb) [file 12890_2017_528_MOESM5_ESM.pdf]
